# Total Brain Volumetric Measures and Schizophrenia Risk: A Two-Sample Mendelian Randomization Study

**DOI:** 10.3389/fgene.2022.782476

**Published:** 2022-03-31

**Authors:** Dan Zhu, Chunyang Wang, Lining Guo, Daojun Si, Mengge Liu, Mengjing Cai, Lin Ma, Dianxun Fu, Jilian Fu, Junping Wang, Feng Liu

**Affiliations:** ^1^ Department of Radiology, Tianjin Medical University General Hospital Airport Hospital, Tianjin, China; ^2^ Department of Radiology and Tianjin Key Laboratory of Functional Imaging, Tianjin Medical University General Hospital, Tianjin, China; ^3^ Department of Scientific Research, Tianjin Medical University General Hospital, Tianjin, China; ^4^ National Supercomputer Center in Tianjin, Tianjin, China

**Keywords:** schizophrenia, total brain volumetric measures, genetic, causality, Mendelian randomization

## Abstract

Schizophrenia (SCZ) is an idiopathic psychiatric disorder with a heritable component and a substantial public health impact. Although abnormalities in total brain volumetric measures (TBVMs) have been found in patients with SCZ, it is still unknown whether these abnormalities have a causal effect on the risk of SCZ. Here, we performed a Mendelian randomization (MR) study to investigate the possible causal associations between each TBVM and SCZ risk. Specifically, genome-wide association study (GWAS) summary statistics of total gray matter volume, total white matter volume, total cerebrospinal fluid volume, and total brain volume were obtained from the United Kingdom Biobank database (33,224 individuals), and SCZ GWAS summary statistics were provided by the Psychiatric Genomics Consortium (150,064 individuals). The main MR analysis was conducted using the inverse variance weighted method, and other MR methods, including MR-Egger, weighted median, simple mode, and weighted mode methods, were performed to assess the robustness of our findings. For pleiotropy analysis, we employed three approaches: MR-Egger intercept, MR-PRESSO, and heterogeneity tests. No TBVM was causally associated with SCZ risk according to the MR results, and no significant pleiotropy or heterogeneity was found for instrumental variables. Taken together, this study suggested that alterations in TBVMs were not causally associated with the risk of SCZ.

## Introduction

Schizophrenia (SCZ) is one of the most serious mental disorders; it has a high disability rate worldwide and has brought heavy economic burdens and life pressure to families and society (Mueser and McGurk, 2004). SCZ has been shown to have a high rate of heritability (60–80%), much of which is attributable to common risk alleles, suggesting that the genome-wide association study (GWAS) can enhance our understanding of the etiology of SCZ (Kahn et al., 2015). The GWAS has revealed that single-nucleotide polymorphisms (SNPs) at novel loci confer risk for SCZ, and these results have been obtained by enlarging sample sizes and incorporating more ethnicities (Ripke et al., 2013; Schizophrenia Working Group of the Psychiatric Genomics Consortium, 2014; Lam et al., 2019).

In addition to the genetic basis, substantial efforts have been made in the past decade to elucidate the neural basis of SCZ by using neuroimaging techniques (Kahn et al., 2015). Neuroimaging measures can be considered as endophenotypes, which are quantitative indicators of brain structure or function that index genetic liability for neuropsychiatric disorders (Meyer-Lindenberg and Weinberger, 2006). Compared to neuropsychiatric disorders, endophenotypes are hypothesized to have less polygenicity, have a greater effect size of susceptible SNPs, and require smaller sample sizes to discover the SNPs (Gottesman and Gould, 2003; Meyer-Lindenberg and Weinberger, 2006). A number of studies have reported alterations in total brain volumetric measures (TBVMs), such as total gray matter volume (TGMV), total white matter volume (TWMV), total cerebrospinal fluid volume (TCSFV), and total brain volume (TBV), in patients with SCZ. For example, Haijma et al. (2013) conducted a meta-analysis on TBVMs in more than 18,000 patients and controls, demonstrating a significant reduction in intracranial volume (ICV, sum of TGMV, TWMV, and TCSFV) and TBV (sum of TGMV and TWMV) and an increase in TCSFV in SCZ patients. In addition, progressive decreases in TBV and ventricular expansions (increased in TCSFV) were found in longitudinal studies of SCZ (Kempton et al., 2010; Olabi et al., 2011). However, all these findings were based on observational studies, which may be limited by the possibility of confounding factors and reserve causation; thus, it is still unknown whether TBVM alterations have a causal effect on the risk of SCZ.

Mendelian randomization (MR) is an epidemiological approach that could overcome the limitations in observation studies by using genetic variants associated with exposure as instrumental variables to uncover the causal relationship between an exposure and an outcome (Lawlor et al., 2008). In addition, MR can control the confounding factors and reverse causation that are usually encountered in observation studies. To date, MR has been successfully applied to assess causal relationships in pioneer studies of neuropsychiatric diseases (Hartwig et al., 2017a; Liu et al., 2018; Vaucher et al., 2018; He et al., 2020; Wang et al., 2020; Zhang et al., 2020). For instance, Hartwig et al. found a protective effect of C-reactive protein and a risk-increasing effect of soluble interleukin-6 receptor on SCZ risk (Hartwig et al., 2017a). Vaucher et al. reported that the use of cannabis was causally associated with an increased risk of SCZ (Vaucher et al., 2018). Therefore, in this study, by leveraging data from the largest GWAS summary statistics on both TBVMs and SCZ, we performed a two-sample MR study to estimate the causal effect of TBVMs, including TGMV, TWMV, TCSFV, and TBV, on the risk of SCZ.

## Materials and Methods

### Study Design

**FIGURE 1:**
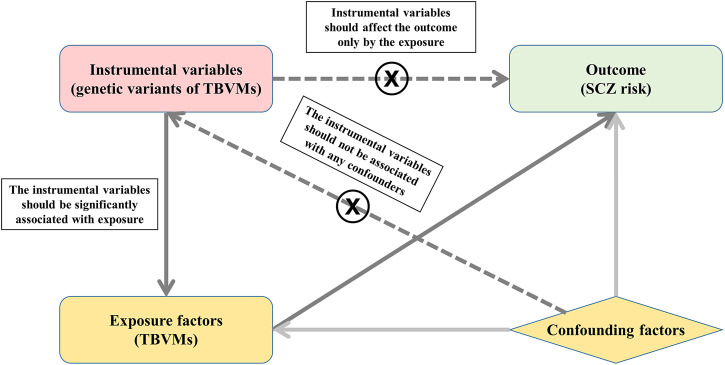
Study design based on MR principal assumptions. In this study, MR is based on the publicly available GWAS summary datasets in TBVMs and SCZ. Specifically, the genetic variants that are significantly associated with TBVMs were used as the instrumental variables to examine the causal influence of TBVMs on SCZ risk. Abbreviations: SCZ, Schizophrenia; TBVMs, total brain volumetric measures.

MR is an approach that uses genetic variants as instrumental variables to investigate the causal relationship between exposures and outcomes, which should satisfy three principal assumptions: 1) the instrumental variables should be significantly associated with exposure; 2) the instrumental variables should not be associated with any confounders; and 3) the instrumental variables should affect the risk of the outcome only by the exposure. The second and third assumptions are also considered independent of pleiotropy. In this study, MR is based on the publicly available GWAS summary datasets of TBVMs (Smith et al., 2021) and SCZ (Schizophrenia Working Group of the Psychiatric Genomics Consortium, 2014), and all subjects provided informed consent in the original studies. Specifically, the genetic variants that were significantly associated with TBVMs were used as instrumental variables to examine the causal influence of TBVMs on SCZ risk (Figure 1).

### Total Brain Volumetric Measure Genome-Wide Association Study Dataset

The GWAS summary data of TBVMs, including TGMV, TWMV, TCSFV, and TBV, were downloaded from an open resource named the Oxford Brain Imaging Genetics (BIG40) web server (https://open.win.ox.ac.uk/ukbiobank/big40/), which included GWAS summary statistics with 33,224 individuals in the United Kingdom Biobank (Smith et al., 2021). The genome-wide significance threshold was set at *p* < 5 × 10^−8^ in the discovery cohort (*N* = 22,138) and *p* < 0.05 in the replication cohort (*N* = 11,086). Only SNPs that met the significance level in both cohorts were used as instrumental variables in MR analyses, and these SNPs were independent and had no linkage disequilibrium, as described in the original studies (Elliott et al., 2018; Smith et al., 2021). Detailed information about the instrumental variables of TGMV, TWMV, TCSFV, and TBV is shown in Supplementary Tables S1-S4.

### Genome-Wide Association Study of Schizophrenia

GWAS summary data regarding SCZ were downloaded from a meta-analysis provided by the Schizophrenia Working Group of Psychiatric Genomics Consortium (https://www.med.unc.edu/pgc/pgc-workgroups/schizophrenia/), including 36,989 cases and 113,075 controls of predominantly European ancestry without population stratification. In total, 128 significant associations in 108 genetic loci were identified (Schizophrenia Working Group of the Psychiatric Genomics Consortium, 2014).

### Pleiotropy Analysis

Comprehensive pleiotropy analyses were performed to assure that instrumental variables met the MR assumptions. First, an MR-Egger intercept test was performed to evaluate the potential pleiotropic associations of the instrumental variables with known and unknown confounders (Bowden et al., 2015; Bowden et al., 2016; Burgess and Thompson, 2017). Second, an MR pleiotropy residual sum and outlier (MR-PRESSO) analysis was carried out to detect horizontal pleiotropy (i.e., MR-PRESSO global test) (Verbanck et al., 2018). Heterogeneity across instrumental variables is also an indicator of pleiotropy. Thus, Cochran’s *Q* test and *I* ^2^ statistic were calculated to estimate the heterogeneity (Sun et al., 2021). Specifically, Cochran’s *Q* test is a conventional test for heterogeneity and approximately follows a chi-square distribution with *n*-1 degrees of freedom (here, *n* is the number of instrumental variables). The *I* ^2^ index is another measure to quantify heterogeneity, which divides the difference between the *Q* statistic and its degrees of freedom by the *Q* statistic itself and then multiplies by 100. The value of the *I* ^2^ index ranges from 0 to 100%, with 0%–25%, 25%–50%, 50%–75%, and 75%–100% representing low, moderate, large, and extreme heterogeneity, respectively (Liu et al., 2013; He et al., 2020). The significance threshold of all the MR-Egger intercept, MR-PRESSO, and Cochran’s *Q* tests was set at *p* < 0.05.

### Aligning Effect Alleles With Exposure and Outcome

The effect alleles of the instrumental variables were adjusted to be associated with increased TBVMs (i.e., the effect estimates of SNPs were larger than zero). Subsequently, the effect alleles of these genetic variants were aligned to be consistent with the effect alleles in the SCZ GWAS dataset. If the instrumental SNPs were not available in the outcome dataset, a proxy SNP that was in high linkage disequilibrium (*r* ^*2*^ > 0.8) with the requested SNP was searched instead with the online tool SNiPA (https://snipa.helmholtz-muenchen.de/snipa3/index.php) (Arnold et al., 2015).

### Two-Sample Mendelian Randomization Analysis

The inverse variance weighted (IVW) method was employed to estimate the causal effects of TGMV, TWMV, TCSFV, and TBV on SCZ risk. Specifically, for each TBVM, the effect estimates of each instrumental variable on TBVMs and SCZ were extracted, and Wald estimates and their standard errors were then calculated (Burgess et al., 2017b). The Wald estimates of all the instrumental variables were combined with a weighted mean using inverse variance weights. The significance threshold of the associations between exposures and outcomes was set at *p* < 0.05.

### Power Analysis

For each TBVM, the proportion of variance explained by each instrumental variable ( $R^{2}$ ) was calculated using the following formula:

$$R^{2}=\frac{2\times MAF\times \left(1-MAF\right)\times \beta^{2}}{2\times MAF\times \left(1-MAF\right)\times \beta^{2}+2\times MAF\times \left(1-MAF\right)\times N\times se{\left(\beta \right)}^{2}}$$

where MAF represents the minor allele frequency for a given SNP, *β* represents the effect size associated with the TBVM for a given SNP, se(β) represents the standard error of the effect size associated with the exposure for a given SNP, and *N* represents the sample size of the exposure GWAS data.

Then, the strength of instrument variables can be measured by *F*-statistics, which were calculated based on the following equation:

$$F=\frac{R^{2}\times \left(N-2\right)}{1-R^{2}}$$

where *R* ^2^ is the proportion of the variance explained by each SNP, and *N* represents the sample size of the exposure GWAS data. To minimize weak instrument bias, SNPs with *F*-statistics > 10 were retained for subsequent analyses (Lawlor et al., 2008).

### Sensitivity Analysis

A series of sensitivity analyses were conducted to validate the robustness of the results. First, four different MR methods including MR-Egger, weighted median, simple mode, and weighted mode methods were performed to estimate the causal effect of TBVMs on SCZ risk. Specifically, the MR-Egger method allows all variants to have pleiotropic effects and can provide a consistent estimate of the causal effect under a weaker instrument strength independent of direct effects (InSIDE) assumption (Burgess and Thompson, 2017); the weighted median method can provide valid causal estimates even if up to 50% of instruments are not valid (Bowden et al., 2016); and the model-based methods (i.e., simple mode and weighted mode) use the causal effect estimates for individual SNPs to form clusters, and the causal effect is estimated in the largest cluster of SNPs (Hartwig et al., 2017b). Second, a leave-one-out sensitivity analysis was carried out to identify SNPs that could potentially bias the causal relationship. To this aim, by sequentially removing each SNP, we estimated the relationship between the remaining SNPs and the risk of SCZ using the IVW method. Finally, reverse causation bias may occur when the outcome variable is at an earlier time point (i.e., the risk of SCZ causally influences the changes of each TBVM). Therefore, we also tested the possibility of reverse causation by treating the risk of SCZ as an exposure and each TBVM as an outcome. Specifically, the instrumental variables were the significant genetic variants associated with SCZ risk, and the same procedures as the main analyses were used to perform reverse MR causality detection.

All statistical analyses were conducted using *R* version 4.0.4 (R Foundation for Statistical Computing, Vienna, Austria) using the packages of “TwoSampleMR” (Hemani et al., 2018b) and “MR-PRESSO” (Verbanck et al., 2018).

## Results

### Association of Total Brain Volumetric Measure Variants With Schizophrenia

Only two genetic variants without linkage disequilibrium were found to be associated with TGMV, and their summary statistics were extracted from SCZ GWAS data for MR analyses (Supplementary Table S1). Of the five genetic variants associated with TWMV, rs742396 was a palindromic SNP. Thus, we deleted it in the subsequent MR analyses (Supplementary Table S2). Seven genetic variants were associated with TCSFV. All seven instrumental SNPs were located on different chromosomes and were not in linkage disequilibrium with each other. However, rs4843550 is a palindromic SNP and was removed from the subsequent MR analyses. The summary statistics for these TCSFV variants are shown in Supplementary Table S3. Of the five genetic variants associated with TBV, the summary statistics for the four variants could be extracted from the SCZ GWAS data. The SNP rs2732714 was not available in the SCZ GWAS data, therefore, we used the information of its proxy SNP rs113138968, which was in high linkage disequilibrium (*r* ^*2*^ > 0.8), to perform the following analyses. All five instrumental SNPs were not in linkage disequilibrium with each other, and none of them were palindromic SNPs. Detailed information about these five instrumental SNPs is shown in Supplementary Table S4.

### Pleiotropy Analysis

Both the MR-Egger intercept test and MR-PRESSO test showed no significant pleiotropy for the genetic variants of TBVMs (all *p*s > 0.05). Furthermore, Cochran’s *Q* test and *I* ^*2*^ statistic revealed no significant heterogeneity for these SNPs (Supplementary Table S5).

### Two-Sample Mendelian randomization Analysis

We performed a two-sample MR analysis by using genetic variants from TGMV, TWMV, TCSFV, and TBV as instrumental variables. As shown in Table 1, we did not find any causal influence on the risk of SCZ with the IVW method (*p* > 0.05).

**TABLE 1:** Results of the causal effect of TBVMs on SCZ risk.

MR methods	TGMV			TWMV				TCSFV			TBV	
	BETA	SE	*p* value	BETA	SE	*p* value	BETA	SE	*p* value	BETA	SE	*p* value
IVW	0.085	0.163	0.601	0.062	0.132	0.636	-0.089	0.089	0.315	0.114	0.090	0.202
MR-Egger	—	—	—	0.004	0.831	0.997	-0.646	0.220	0.042	1.204	0.914	0.279
Weighted median	—	—	—	0.025	0.122	0.841	0.012	0.088	0.089	0.081	0.094	0.384
Simple mode	—	—	—	0.003	0.178	0.986	0.064	0.127	0.635	0.070	0.141	0.649
Weighted mode	—	—	—	0.001	0.159	0.997	0.060	0.118	0.631	0.064	0.145	0.681

Abbreviations: BETA, regression coefficient; IVW, inverse variance weighted; MR, Mendelian randomization; SE, standard error; TBV, total brain volume; TCSFV, total cerebrospinal fluid volume; TGMV, total gray matter volume; TWMV, total white matter volume.

Notably, only the IVW method worked when there were two instrumental variables in TGMV-SCZ MR analysis.

### Power Analysis

The explained variances (*R* ^2^) and *F*-statistics of each instrumental variable are shown in Supplementary Tables S1-S4, and the *F*-statistics of each instrumental variable were larger than 10, indicating no weak instrumental bias among these variables.

### Sensitivity Analysis

**FIGURE 2:**
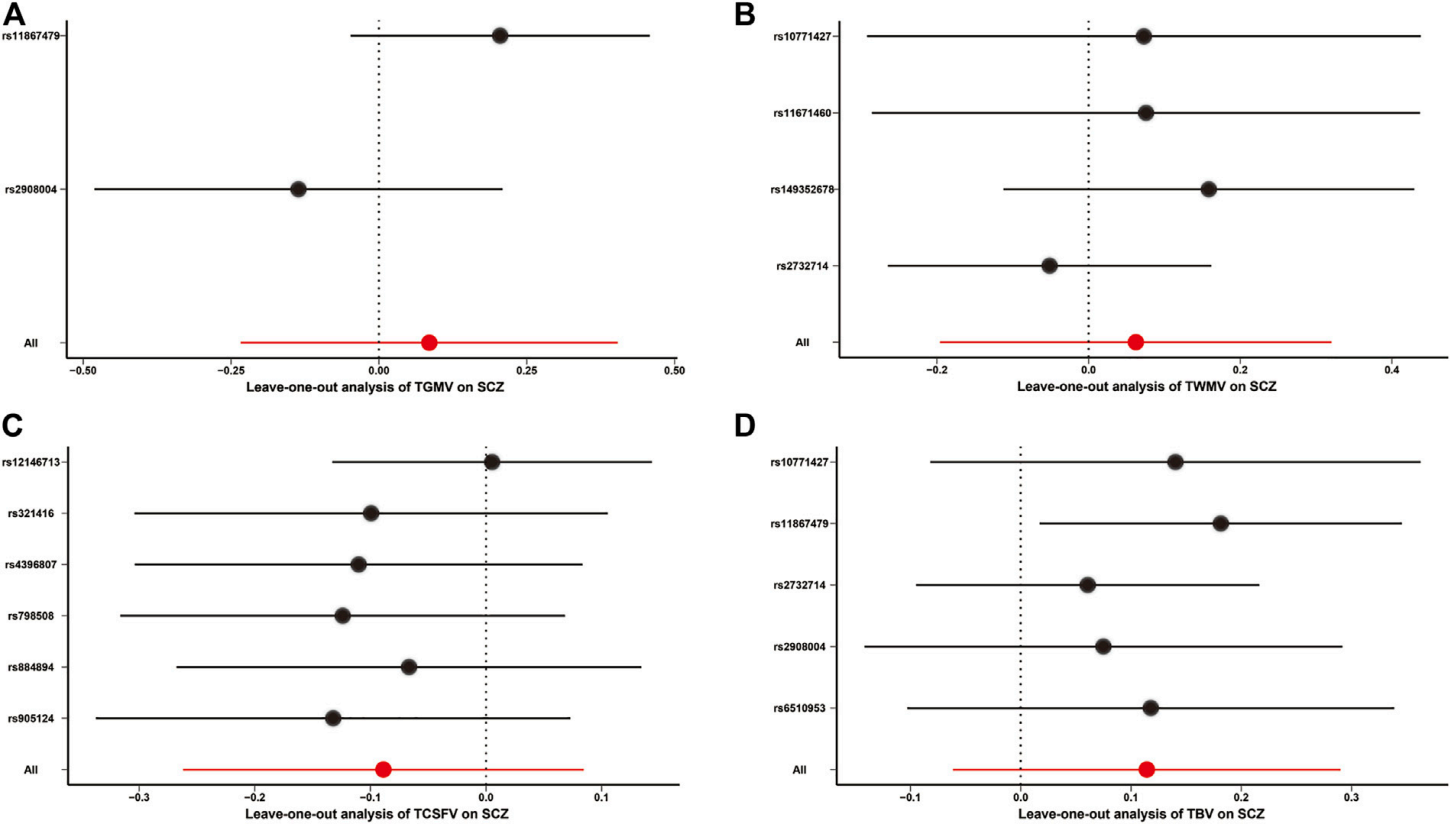
Leave-one-out analysis for MR causality analysis between TBVMs and SCZ risk. **(A)**. Leave-one-out analysis for MR causality analysis between TGMV and SCZ risk. **(B)**. Leave-one-out analysis for MR causality analysis between TWMV and SCZ risk. **(C)**. Leave-one-out analysis for MR causality analysis between TCSFV and SCZ risk. **(D)**. Leave-one-out analysis for MR causality analysis between TBV and SCZ risk. The red points and red lines represent the BETA and 95% confidence interval in MR analyses, while the black points and black lines represent the BETA and 95% confidence interval after removing each SNP sequentially. Of note, only the IVW method was used in the leave-one-out sensitivity analysis. Abbreviations: SCZ, Schizophrenia; TBV, total brain volume; TCSFV, total cerebrospinal fluid volume; TGMV, total gray matter volume; TWMV, total white matter volume.

All other MR approaches, including the MR-Egger, weighted median, simple mode, and weighted mode methods, did not identify any significant causal effects of TGMV, TWMV, and TBV on the risk of SCZ (Table 1). Although TCSFV was found to be causally associated with the risk of SCZ when using the MR-Egger method (*BETA* = 0.646, *SE* = 0.220, *p value* = 0.042), this result was not validated by other methods. In leave-one-out sensitivity analyses, no genetic variants could significantly affect the MR estimates (Figure 2). For the reverse MR causality analysis, 111 leading SNPs associated with SCZ risk were extracted from the GWAS summary data of TBVMs. Among them, ten palindromic SNPs were removed, and the remaining 101 SNPs were retained for subsequent analyses (Supplementary Table S6). All the MR methods indicated that there was no causal influence of any TBVM on SCZ risk (Supplementary Table S7).

## Discussion

SCZ is a chronic, complex mental disorder characterized by an array of symptoms, including delusions, hallucinations, disorganized speech, and impaired cognitive ability, that typically emerges in late adolescence and early adulthood (Mueser and McGurk, 2004; Sheffield and Barch, 2016; Marder and Cannon, 2019; McCutcheon et al., 2020). Several lines of evidence have suggested that structural brain abnormalities play an important role in the pathology of SCZ (Okada et al., 2016; Zhao et al., 2018; Kuo and Pogue-Geile, 2019). Using neuroimaging methods, some researchers found TBVM abnormalities in patients with SCZ relative to age-matched healthy controls (Staal et al., 1998; Haijma et al., 2013), and progressive reductions in TBVMs might be associated with disease progression (Kempton et al., 2010). However, evidence has pointed toward the possibility that antipsychotic drugs might have an effect on TBVM alterations (Olabi et al., 2011; Guo et al., 2015; Emsley et al., 2017). In addition, as a risk factor for SCZ, experience with cannabis use could also lead to brain structural alterations (Kumra et al., 2012; Rapp et al., 2012; Navarri et al., 2022). Hence, the causality between the changes in TBVMs and the risk of SCZ remains largely unclear.

In this study, we aimed to explore whether there is a causal effect of changes in TBVMs on the risk of SCZ by using MR, one of the powerful genetic-epidemiological approaches. Here, we used four reliable TBVMs (TGMV, TWMV, TCSFV, and TBV) derived from structural neuroimaging data. Specifically, genetic variants of TGMV, TWMV, TCSFV, and TBV without any pleiotropy and heterogeneity were selected as the instrumental variables, and five MR methods were used to ensure the reliability of the results. Different from the observational studies, no significant result was found using MR between any TBVMs and SCZ risk. The possible explanations for the difference are as follows: 1) the substantial brain structural heterogeneity exists across the individuals with SCZ (Alnæs et al., 2019). The changes in TBVMs might not be a sensitive risk factor for SCZ, since alterations (increase or decrease) in the volume of some specific brain regions have been reported in patients with SCZ (Kuo and Pogue-Geile, 2019); 2) some observational studies showed that the decrease in TBVMs in SCZ might be the result of antipsychotics, aging, or other unknown confounders (Kumra et al., 2012; Emsley et al., 2017); and 3) SCZ is a cognitive and behavioral dysfunction with complex symptoms (Sheffield and Barch, 2016), the onset of which might be linked to functional abnormalities rather than structural abnormalities of the brain. Hence, more attention should be devoted to the changes in specific brain region volumes by removing the effects of antipsychotics and aging and the functional neural mechanisms of SCZ.

Our study design has many advantages. First, the exposure and outcome datasets were from a large-scale GWAS of TBVMs (*N* = 33,224) and SCZ (36,989 cases and 113,075 controls). The large sample sizes of GWAS typically led to higher levels of statistical power (van der Sluis et al., 2013). Second, we utilized independent SNPs as the instrumental variables in each MR analysis, which could effectively avoid the influence caused by linkage disequilibrium. Third, a series of pleiotropy and sensitivity analyses based on different principles and assumptions were carried out to detect pleiotropy and heterogeneity to ensure that the instrumental variables we used here were reliable (Burgess et al., 2017a; Hemani et al., 2018a). Finally, to increase the robustness of the MR results, different methods were applied to investigate the causal relationship between the exposures and the outcomes. Assessing the causal relationship by using a variety of methods is more reliable because the different MR methods we used here were based on the different assumptions (Burgess and Thompson, 2017).

Some limitations needed to be addressed in this study. First, the subjects from the outcome GWAS dataset were of transancestral descent (both European and East Asian); however, the subjects from the TBVM GWAS dataset were of pure European descent. Population stratification might have a potential confounding effect on the causal estimate. Second, although a series of statistical methods were used to identify pleiotropy, it is impossible to fully remove all pleiotropy in MR studies. Third, the instrumental variables of TBVMs were obtained from United Kingdom Biobank GWAS summary data. The participants in the United Kingdom Biobank were aged from 45 to 81 years (Smith et al., 2021), which is not the typical age of onset for SCZ (Howard et al., 2000). The genetic variants determining TBVMs in childhood and/or adolescence may differ from those determining TBVMs in adulthood used in this study. Therefore, it would be better to use instrumental variables from large-scale TBVMs GWAS data in childhood and/or adolescence that are not publicly available to date. Fourth, the generalized summary-based MR (GSMR) method is also a popular MR approach to assess the causal association between exposure and outcome (Zhu et al., 2018). The rule of thumb advises that the application of GSMR requires ten or more independent genome-wide significant SNPs, but there were fewer than ten instrumental variables used in each two-sample MR analysis in our study, especially those of TGMV. Thus, we could not use the GSMR method to test the causal associations of TBVMs with SCZ risk. Finally, ICV is also an important TBVM, and alterations in ICV were found in SCZ patients (Haijma et al., 2013). We did not investigate the causal relationship between ICV and SCZ risk in this study because there are no GWAS summary data of ICV in the United Kingdom Biobank database.

## Conclusion

In conclusion, although the previous neuroimaging studies showed the changes in TBVMs in patients with SCZ, our MR results demonstrated that there was no causal relationship between alterations in TBVMs and the risk of SCZ at the genetic level. Further studies with independent data are warranted to confirm these findings.

## Data Availability

Publicly available datasets were analyzed in this study. These data can be found here: the brain volume GWAS summary data were downloaded from the Oxford Brain Imaging Genetics (BIG40) web server (https://open.win.ox.ac.uk/ukbiobank/big40/). GWAS summary data of SCZ were provided by the Schizophrenia Working Group of Psychiatric Genomics Consortium (https://www.med.unc.edu/pgc/pgc-workgroups/SCZ/).
